# Chemical composition, energy and nutritional values, digestibility and functional properties of defatted flour, protein concentrates and isolates from *Carbula marginella* (Hemiptera: Pentatomidae) and *Cirina butyrospermi* (Lepidoptera: Saturniidae)

**DOI:** 10.1186/s13065-021-00772-z

**Published:** 2021-08-09

**Authors:** Aminata Séré, Adjima Bougma, Bazoin Sylvain Raoul Bazié, Esther Traoré, Charles Parkouda, Olivier Gnankiné, Imael Henri Nestor Bassolé

**Affiliations:** 1 Department of Biochemistry Microbiology, Joseph KI-ZERBO University, 03 B.P. 7021, Ouagadougou 03, Burkina Faso; 2 Department of Food Technology, National Center for Scientific and Technological Research, 03 B.P. 7047, Ouagadougou 03, Burkina Faso; 3 Department of Animal Biology and Physiology, Joseph KI-ZERBO University, 03 B.P. 7021, Ouagadougou 03, Burkina Faso

**Keywords:** Edible insects, Defatted flour, Isolate, Concentrate, Nutritional value, Digestibility, Functional property

## Abstract

Edible insects constitute a potential source of alternative proteins as a food supplement. The present study aimed to investigate the chemical composition, energy and nutritional values, the digestibility and functional properties of *Carbula marginella* (Thunberg) and *Cirina butyrospermi* (Vuillet) defatted flour, protein concentrates, and isolates. *Carbula marginella* has shown the highest content of protein (41.44%), lipid (51.92%), calcium (33.92 mg/100 g) and sodium (185.84 mg/100 g) while the highest contents of carbohydrate (34.54%), ash (4.77%), iron (31.27 mg/100 g), magnesium (150.09 mg/100 g), and potassium (1277 mg/100 g) have been observed for *C. butyrospermi.* Linoleic (30.23%), palmitic (27.54%), oleic (26.41%) and stearic (8.90%) acids were the most dominant fatty acids found in *C. marginella*. *Cirina butyrospermi* was characterized by high levels of oleic (27.01%), stearic (21.02%), linolenic (20.42%), palmitic (13.06%), and linoleic (8.01%) acids. Protein and essential amino acid contents of the protein isolates in both insect species were 1.7–2 times higher than that of their defatted flours. The protein isolate of *C. marginella* exhibited the highest protein digestibility (87.63%), while the highest fat absorption capacity (8.84 g/g) and foaming capacity (48.40%) have been obtained from the protein isolate of *C. butyrospermi*. These findings indicate that the protein concentrates and isolates of *C. marginella* and *C. butyrospermi* have great potential for industrial applications.

## Introduction

Proteins are major nutritional components providing both essential and non-essential amino acids to the human body [1]. The current world demand for dietary proteins, estimated at 202 billion tons, is projected to reach around 404 billion tons in 2050 [2]. In addition to their nutritional properties, proteins have several functional properties (water absorption capacity, fat absorption capacity, emulsifying capacity, foaming capacity) in a few food products [3]. The isolation of protein-rich fractions from both animal and plant sources has grown in importance in recent years. The main sources for protein extractions are cereals, pulses, tubers, oilseeds, milk, meat, and fish [2, 4, 5].

Edible insects have been described as sources of protein with an average content ranging from 35.34 to 61.32% [6]. Species from the order of Orthoptera displayed high protein content ranging from 58.90 to 77.13% [6]. Edible insects of Diptera order can cover the adults’ requirements for methionine and methionine + cysteine which are limited amino acids of cereal and pulse seeds [6]. However, edible insect protein has a lower digestibility compared to casein [7]. But, insect protein digestibility can be improved by removing the chitin exoskeleton which lowers its level of digestibility [8]. Insect protein functional properties can be improved by their extraction, thus making them suitable food supplements [9, 10]. *Carbula marginella* and *Cirina butyrospermi* are both the most preferred edible insects in Burkina Faso[11]. *Cirina butyrospermi* is a pest from the shea butter tree, belonging to the Lepidoptera order. In the southern Sudan area, it is mainly consumed fried or as ingredients in various sauces by the Bobo, Guin, Sambla, Senoufo, and Turka ethnic groups [11]. It is exclusively found in the south Sudanian zone under specific rainfall (900 to 1000 mm) and humidity conditions (70–85%) [12]. *Cirina butyrospermi* is also consumed in some countries such as Botswana, Cote d’Ivoire, Democratic Republic of Congo, Ghana, Mali, Mozambique, Namibia, Nigeria, South Africa, Togo, and Zambia [11, 13–15]. *Cirina butyrospermi*, being specifically rich in protein (55.41–62.74%) and fat (14.51–28.71%), is highly nutritious [16, 17]. It contains all essential amino acids and also has a high content of linolenic acid [16, 17].

*Carbula marginella* belongs to the Hemiptera (true bugs) order, consumed in the northern Sudanian zone by the Mossi and Fulani ethnic groups. It is found in cave holes in this area during the dry season (October to January) and it is consumed roasted [11]. However, to the best of our knowledge, there are no published data on the chemical composition, nutritional value, digestibility, and functional properties of defatted flour, protein concentrates, and isolates of both *C. marginella* and *C. butyrospermi*. The aim of the present study was to compare the chemical composition, energy and nutritional values, digestibility, and functional properties of defatted flour, protein concentrates, and isolates of *C. marginella* and *C. butyrospermi*, two common species of insects used as food in Burkina Faso.

## Materials and methods

### Materials

**Fig. 1:**
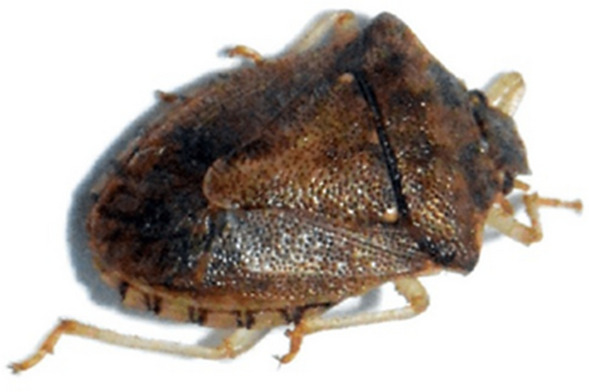
*Carbula marginella*

**Fig. 2:**
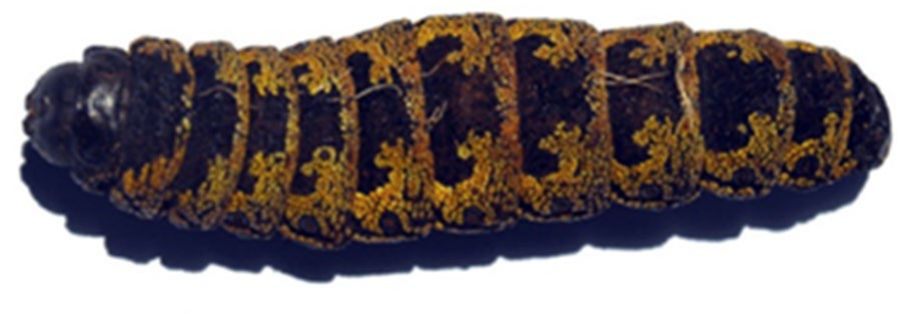
*Cirina butyrospermi*

*Carbula marginella* adults (Fig. 1) were collected in the village of Boudtenga (12° 29′ 11′′ N; 1° 15′ 57′′ W) in the North Sudanian zone in December 2016. *C. butyrospermi* (Fig. 2), at the fourth stage of its development (larval stage), was collected in the South Sudanian zone from July to August 2015 and 2017 in the village of Koumi (N 11° 07′ 54.7′′, W 004° 25′ 41.5′′). The insects were immediately placed in cooler boxes containing ice and brought to the laboratory. Specimen were identified at the Department of Environment and Forests using the Scholtz classification [18]. Samples were cleaned and the inedible parts were removed. Then, they were washed with distilled water and dried for 24 h in an oven at 40–50 °C.

Alpha-chymotrypsin (MP Biomedicals, USA), nitric acid (Carlo Erba, France, 96%), boric acid (Sigma-Aldrich, USA, 98%), ethanol (Chromasolv, absolute, for HPLC, Sigma-Aldrich, Germany), *n*-hexane (Chromasolv, Germany), hydrochloric acid (Carlo Erba, France, 37%), Kjeldahl catalyst (Carlo Erba, Germany), phenylisothiocyanate (Thermo Scientific, USA), methanol (HPLC Gradient Grade for free amino acids analysis, Prolabo Chemicals, France), Pico Tag diluent (Waters, USA), sodium hydroxide (Carlo Erbo, France), triethylamine (Sigma-Aldrich, Belgium, 99%) and trypsin from bovine pancreas (Sigma Aldrich, USA) were used.

### Preparation of protein isolate

The protein isolate was extracted according to the Wolf method with minor modifications [19]. Briefly, the defatted flour was stirred for 2 h at room temperature (about 25 °C) with de-ionized water, pH adjusted to 11.0 with 1 N NaOH [water: flour ratio, 1:20 (w/v)]. The slurry was centrifuged at 10,000*g* for 30 min at 4 °C. The pellet was re-dissolved with adjusted pH de-ionized water, as described above, and cold-centrifuged again. The supernatants were mixed together, the pH was adjusted to 4.5 with 1 N HCl. The mixture was then kept for 2 h at room temperature and subsequently centrifuged at 10,000*g* for 30 min at 4 °C. The precipitate was washed with de-ionized water and re-dissolved in de-ionized water. The pH was neutralized to 7.0 with 1 N NaOH at room temperature, and then freeze-dried.

### Preparation of protein concentrate

The protein concentrate was prepared according to the process described by Wolf with minor modifications [19]. The defatted flour was stirred for 1 h at room temperature (about 25 °C). The suspension was filtered and the residues were air-dried in a fume hood. Residues were dispersed in de-ionized water (1:20, w/v) at room temperature. The pH was adjusted to 4.5 by the addition of 1 N HCl. The slurry was stirred for 2 h and centrifuged (10,000*g*, 30 min, 4 °C). The precipitate was washed with de-ionized water, re-dissolved in de-ionized water, and the pH was neutralized to 7.0 with 1 N NaOH at room temperature, and then freeze-dried.

### Proximate analysis and energy value

Moisture, fat, protein, and ash contents were determined according to AOAC official methods 950.46, 960.39, 979.09, and 920.153, respectively [20]. Moisture percentage was calculated by drying the sample in an oven at 105 °C for 3 h. Fat percentage was calculated by drying fats after extraction in a Soxhlet using petroleum ether. Crude protein was determined by the Kjeldahl method and the total protein content was calculated as the amount of total determined N multiplied by the nitrogen to a protein conversion factor of 6.25. Ash percentage was calculated by combusting the samples at 550 °C for 4 h in a porcelain crucible placed in a muffle furnace. The Atwater conversion factors were used to calculate the energy value [21]. All the analyses were performed in triplicate and expressed as mean ± standard deviation.

### Mineral compositions

To determine the mineral content of insect flour, 5.0 g of powdered samples were incinerated in a furnace at 550 °C and the residues were dissolved in 50 mL of 0.5 M HNO_3_ solution. The concentrations of calcium (Ca), iron (Fe), magnesium (Mg), potassium (K), sodium (Na), and zinc (Zn) were determined according to AOAC official method 999.11 [20]. The analyses were performed in triplicate and expressed as mean ± standard deviation.

### Fatty acid methyl ester preparation and gas chromatography (GC) analysis

The fatty acid (FA) compositions of the oils were determined following the International Union of Pure and Applied Chemistry (IUPAC) method [22]. Fatty acid methyl esters (FAME) were prepared following the method described by Khan [23]. About 50 mg of the oil samples were weighed into a test tube and dissolved with 1 mL hexane by vortexing for 30 s. Then, 2 mL of 4 M methanolic KOH was added into the test tube which was vortexed again for 30 s. The mixture was placed in the React-Therm module for 30 min at 50 °C and cooled at room temperature. 1 mL of de-ionized water was then added. An aliquot of the organic layer was transferred into a vial for injection. FA compositions were determined using a Gas Chromatograph-FID (Agilent Technologies 6890 N, Palo Alto, CA, US) with a DB23 capillary column (60 m ID: 0.25 mm, film: 0.25 μm, J&W Scientific Co., CA, USA). The working conditions of Gas Chromatography (GC) were as follows: 1 µL injection volume, 1:50 injector split ratio, 1 mL/min flow rate, Nitrogen as a carrier gas, hydrogen (40 mL/min), and dry air (450 mL/min) as detector gases, 200 °C inlet temperature, and 250 °C detector temperature. The oven temperature was in gradient mode programs from 130 to 240 °C. The oven was programmed to operate at 130 °C for 5 min, increased to 170 °C at 5 °C/min, to 215 °C at 1.5 °C/min, to 240 °C at 40 °C/min, and kept constant for an additional 5 min. FA was identified by using a FAME standard mixture (30-components, Supelco, Bellefonte, PA, USA). All determinations were performed in three replicates.

### Protein content and amino acid composition

The profile and amount of amino acids were determined by reverse-phase of High-Performance Liquid Chromatography (HPLC) using the Pico Tag method described by Bidlingmeyer et al. [24]. The samples were defatted with n-hexane and hydrolyzed with 6 N hydrochloric acid. About 0.4 g of the defatted sample was transferred in a bottle with 15 mL of hydrochloric acid and incubated in an oven at 110 °C for 24 h. The sample was then left to cool to room temperature, poured into a 50 mL volumetric flask, completed with Milli-Q water, and mixed. Approximately 1 mL of the diluted and homogenized solution was filtered through a Polytetrafluoroethylene (PTFE) filter of 0.45 μm. An aliquot of 10 µL of the solution was dried under vacuum for 15 min using Pico Tag Workstation. The sample was then re-dissolved in 10 µL of ethanol/water/triethylamine (2:2:1 volume) and replaced again at the Pico Tag Workstation under vacuum dehydration for 15 min. The dehydrated sample was mixed again with 20 µL of derivatization solution incorporated of ethanol/triethylamine/water/phenylisothiocyanate (7:1:1:1 volume). This was kept for 20 min at room temperature and the excess reagent was removed with a vacuum for 45 min [25]. The amino acid derivatives were then separated by HPLC and detected by UV detector at 254 nm after elution through a Pico Tag precolumn [Nova-Pak C18 Guard Column, 60Å, 4 μm, 3.9 mm × 20 mm) and column (C18 PICO’TAG Column Waters (3.9 × 150mm)] according to the conditions described by Bidlingmeyer et al. [24]. The analysis (identification and quantification) of amino acids was then carried out using Empower 2 software (Waters, USA).

### Protein digestibility

The three-enzyme method of Hsu et al. [26] and Satterlee et al. [27] was used. Ten millilitre of an aqueous protein suspension (1 mg per mL distilled water) was equilibrated at 37 °C to pH 8.0. One millilitre of three-enzyme solution (1.61 mg trypsin, 3.96 mg chymotrypsin, and 2.36 mg peptidase per mL) was added to the protein suspension, and after exactly 10 min of incubation, the pH was recorded. The calculation of in-vitro digestibility coefficients has been obtained from:

$${\text{Digestibility}}=4.33+53.21 {\text{X}}$$

X is the Volume of NaOH (mL poured at T = 10 min to maintain the pH at 8.0.

### Functional properties

#### Water absorption capacity (WAC)

Water absorption capacity (WAC) was determined by the method outlined by Diniz and Martin [28] with slight modifications. 0.5 g of defatted flour, protein concentrate, or protein isolate was dispersed in 20 mL of distilled water and stirred with a shaker at 540 rpm for 30 min. Afterward, the dispersion was centrifuged at 8000*g* for 15 min and the precipitate was weighed. Then, WAC was calculated as follows:

$$WAC=\frac{W2-W1}{W}$$

where *W* is the weight of the dry sample (in g), *W*_1_ is the weight of the tube plus the dry sample (in g) and *W*_2_ is the weight of the tube plus the sediment (in g).

#### Fat absorption capacity

Fat absorption capacity (FAC) was determined using the procedure of Haque and Mozaffar with slight modifications [29]. 0.5 g of defatted flour, protein concentrate, or protein isolate was added to 10 mL of vegetable oil and vortexed for 30 s to mix. Afterward, the dispersion was centrifuged at 8000*g* for 15 min and the precipitate was weighed. Then, FAC was calculated as follows:

$$FAC=\frac{F2-F1}{F0}$$

where F_0_ is the weight of the dry sample (in g), F_1_ is the weight of the tube plus the dry sample (in g) and F_2_ is the weight of the tube plus the sediment (in g).

#### Foaming capacity

Foaming capacity (FC) was determined according to the method described by Guo et al. [30]. Twenty milliliters of 1% sample were homogenized in a high shear homogenizer mixer at a speed of 16,000 rpm for 2 min. The whipped sample was immediately transferred into a cylinder. FC was calculated according to the formula:

$$FC\left(\%\right)=\frac{VO-V}{V}$$

where V is the volume before whipping (mL), V0 is the volume after whipping (mL).

### Statistical analysis

The analytical measures were performed at least in triplicate and the results of the different parameters were presented by the means ± standard deviation. Statistical analysis was performed using the XLSTAT software for Windows (XLSTAT 2016.02.27444). A comparison of the means was performed by One-way analysis of variance (ANOVA) followed by Tukey test.

## Result

The fresh matters of both *C. marginella* and *C. butyrospermi* were characterized by high moisture contents of 64.9 ± 1.49 and 73.84 ± 2.86 g/100 g, respectively (Table 1). Significant differences were observed in their proximate composition and energy value (*p* < 0.05). *Carbula marginella* had the highest content of protein (41.44 ± 0.05%), lipid (51.92 ± 0.40%) and energy value (650.23 ± 2.11 Kcal/100 g of dry matter) whereas *C. butyrospermi* exhibited the highest ash (4.77 ± 0.02%) and carbohydrate (34.54 ± 0.29%) contents (Table 1).

**Table. 1:** Moisture, proximate composition on dry basis (g/100 g) and energy value (Kcal/100 g) of *Carbula marginella* and *Cirina butyrospermi*

Parameters	*Carbula marginella*	*Cirina butyrospermi*
Moisture (wet basis)	64.9 ± 1.49^b^	73.84 ± 2.86^a^
Ash	2.34 ± 0.01^b^	4.77 ± 0.02^a^
Crude protein	41.49 ± 0.05^a^	40.81 ± 0.20^b^
Crude fat	51.92 ± 0.40^a^	19.86 ± 0.11^b^
Carbohydrates	4.24 ± 0.34^b^	34.54 ± 0.29^a^
Energy value	650.23 ± 2.11^a^	480.20 ± 0.49^b^

The same superscript letters in each row indicate no significant difference (p-value < 0.05) between the mean values

Fe, Mg, and K contents of *Cirina butyrospermi* were two to threefolds higher than those of *C. marginella. Carbula marginella* had 14 times higher Na content than that of *C. butyrospermi* while for Zn and Ca contents, no significant difference was found between the two edible insects (Table 2).

**Table. 2:** Mineral composition of *Carbula marginella* and *Cirina butyrospermi* expressed as mg/100 g dry matter

Mineral	*Carbula marginella*	*Cirina butyrospermi*
Iron	10.10 ± 0.06^b^	31.27 ± 0.002^a^
Zinc	10.10 ± 0.07^a^	10.00 ± 0.01^a^
Calcium	33.92 ± 0.27^a^	32.01 ± 0.09^b^
Magnesium	74.55 ± 0.14^b^	150.09 ± 0.00^a^
Potassium	362.06 ± 0.25^b^	1277.75 ± 0.01^a^
Sodium	185.84 ± 0.55^a^	13.25 ± 0.01^b^

The same superscript letters in each row indicate no significant difference (p-value < 0.05) between the mean values

The fatty acid compositions of *C. marginella* and *C. butyrospermi* are given in Table 3. *Carbula marginella* and *C. butyrospermi* contained 38.04 and 42.07% saturated fatty acid (SFA), 30.79%, and 29.23% mono-unsaturated fatty acid (MUFA), and 31.13 and 29.01% poly-unsaturated fatty acid (PUFA), respectively. Quantitatively, the most abundant fatty acids in the oil of *C. marginella* were linoleic acid (30.23 ± 0.08%), palmitic acid (27.54 ± 0.14%), and oleic acid (26.41 ± 0.05%). The oil of *C. butyrospermi* was characterized by high contents of oleic acid (27.01 ± 0.68%), stearic acid (21.02 ± 0.26%), linolenic acid (20.42 ± 0.45%), and palmitic acid (13.06 ± 1.32%).

**Table. 3:** Fatty acids profile of the lipid content extracted from *Carbula marginella* and *Cirina Butyrospermi*

Fatty acids (%)	*Carbula marginella*	*Cirina butyrospermi*
Caproic acid	0.18 ± 0.09^b^	0.43 ± 0.02^a^
Capric acid	0.11 ± 0.01^b^	0.79 ± 0.05^a^
Lauric acid	0.08 ± 0.01^b^	0.60 ± 0.03^a^
Myristic acid	0.29 ± 0.01^b^	0.43 ± 0.05^a^
Palmitic acid	27.54 ± 0.14^a^	13.06 ± 1.32^b^
Margaric acid	0.12 ± 0.00^b^	0.89 ± 0.02^a^
Stearic acid	8.90 ± 0.08^b^	21.02 ± 0.26^a^
Arachidinic acid	0.69 ± 0.00^b^	1.54 ± 0.68^a^
Lignoceric acid	0.13 ± 0.02^b^	3.31 ± 0.49^a^
Palmitoleic acid	4.06 ± 0.09^a^	0.83 ± 0.04^b^
Oleic acid	26.41 ± 0.05^a^	27.01 ± 0.68^a^
Gondoic acid	0.32 ± 0.08^b^	0.94 ± 0.03^a^
Linoleic acid	30.23 ± 0.08^a^	8.01 ± 0.08^b^
Linolenic acid	0.79 ± 0.02^b^	20.42 ± 0.45^a^
Docosapentaenoic acid	0.11 ± 0.01^b^	0.57 ± 0.13^a^
SFA	38.04	42.07
MUFA	30.79	29.23
PUFA	31.13	29.01
Total	99.96	99.85

The same superscript letters in each row indicate no significant difference (p-value < 0.05) between the mean values

*SFA* Saturated fatty acids, *MUFA* Monounsaturated fatty acids, *PUFA* Polyunsaturated fatty acids

Significant differences were found in protein contents and amino acid compositions of isolate, concentrate, and defatted flour of *C. marginella* and *C. butyrospermi* (Tables 4 and 5) (*p* < 0.05). The highest protein content was found in the isolate fractions for both *C. butyrospermi* (87.41 ± 0.24%) and *C. marginella* (85.78 ± 0.58%) whereas defatted flours exhibited about half of the isolate content of 40.81 ± 0.20 and 41.49 ± 0.05% for *C. butyrospermi* and *C. marginella*, respectively.

**Table. 4:** Protein contents and amino acid compositions of defatted flour, protein concentrate and isolate of *Carbula marginella* expressed as g/100 g dry matter

Proteins/amino acids	Defatted flour	Protein concentrate	Protein isolate
Proteins	41.49 ± 0.05^c^	54.47 ± 0.92^b^	85.78 ± 0.58^a^
Histidine	1.72 ± 0.07^b^	2.02 ± 0.19^a^	1.39 ± 0.11^c^
Threonine	5.73 ± 1.04^c^	9.45 ± 0.79^a^	7.62 ± 0.23^b^
Valine	2.63 ± 0.2^b^	3.57 ± 0.23^a^	3.82 ± 0.27^a^
Methionine + cysteine	2.10 ± 0.76^a^	1.04 ± 0.01^b^	1.63 ± 0.35^ab^
Isoleucine	2.14 ± 0.36^b^	1.52 ± 0.14^b^	5.24 ± 0.95^a^
Leucine	2.46 ± 0.60^c^	4.03 ± 0.29^b^	6.81 ± 0.00^a^
Lysine	1.05 ± 0.40^c^	4.38 ± 0.53^b^	11.04 ± 0.50^a^
Phenylalanine + tyrosine	2.60 ± 0.25^b^	1.90 ± 0.07^c^	3.89 ± 0.45^a^
Tryptophane	ND	ND	ND
Aspartic acid and asparagine	2.45 ± 0.78^c^	3.68 ± 1.15^b^	10.35 ± 0.00^a^
Glutamic acid and glutamine	2.75 ± 0.50^c^	4.12 ± 0.24^b^	10.97 ± 0.89^a^
Serine	5.23 ± 0.15^a^	3.81 ± 0.38^b^	5.77 ± 0.99^a^
Glycine	4.22 ± 0.73^c^	7.54 ± 0.79^b^	9.41 ± 0.76^a^
Alanine	1.32 ± 0.07^a^	0.75 ± 0.02^b^	1.49 ± 0.18^a^
Arginine	1.54 ± 0.34^c^	2.02 ± 0.04^b^	3.60 ± 0.08^a^
Proline	3.22 ± 0.49^b^	4.54 ± 0.11^a^	2.61 ± 0.62^b^
Essential amino acids	20.43	27.91	41.44
Non-essential amino acids	20.73	26.46	44.20

The same superscript letters in each row indicate no significant difference (p-value < 0.05) between the mean values

*ND* No detected

**Table. 5:** Protein contents and amino acid compositions of defatted flour, protein concentrate and isolate of *Cirina butyrospermi* expressed as g/100 g dry matter

Proteins/amino acids	Defatted flour	Protein concentrate	Protein isolate
Proteins	40.81 ± 0.20^c^	65.62 ± 1.54^b^	87.41 ± 0.24^a^
Histidine	0.96 ± 0.52^a^	1.56 ± 0.37^a^	1.56 ± 0.11^a^
Threonine	2.47 ± 0.19^b^	3.62 ± 0.94^a^	4.18 ± 0.98^a^
Valine	0.36 ± 0.03^b^	0.64 ± 0.1^a^	0.55 ± 0.28^a^
Methionine + cysteine	4.32 ± 0.33^c^	5.46 ± 0.41^b^	7.45 ± 0.56^a^
Isoleucine	3.10 ± 0.15^b^	5.34 ± 1.25^a^	6.51 ± 0.58^a^
Leucine	1.97 ± 0.18^**c**^	2.15 ± 0.36^bc^	4.24 ± 0.59^a^
Lysine	5.38 ± 0.01^**c**^	7.76 ± 0.10^b^	10.22 ± 0.00^a^
Phenylalanine + tyrosine	2.72 ± 0.00^a^	1.75 ± 073^b^	2.53 ± 0.35^ab^
Tryptophan	ND	ND	ND
Aspartic acid + asparagine	3.04 ± 0.00^c^	9.56 ± 0.00^b^	11.51 ± 1.24^a^
Glutamic acid + glutamine	4.38 ± 0.98^c^	7.43 ± 0.93^b^	10.81 ± 1.44^a^
Serine	3.73 ± 0.84^b^	3.35 ± 0.82^b^	6.05 ± 0.42^a^
Glycine	3.04 ± 1.48^c^	6.45 ± 1.6^b^	9.37 ± 0.66^a^
Alanine	2.95 ± 1.08^b^	7.42 ± 1.9^a^	7.14 ± 0.69^a^
Arginine	0.38 ± 0.05^c^	0.78 ± 0.17^ab^	1.08 ± 0.3^a^
Proline	2.17 ± 0.97^b^	2.40 ± 0.68^b^	4.15 ± 0.44^a^
Essential amino acids	21.29	28.26	37.23
Non-essential amino acids	19.68	37.39	50.12

The same superscript letters in each row indicate no significant difference (p-value < 0.05) between the mean values

The content of total essential amino acids of *C. marginella* increased from 20.43 g/100 g dry matter for defatted flour to 27.91 and 41.44 g/100 g dry matter for the protein concentrate and isolate, respectively. Similar increases were observed for *C. butyrospermi* with the values of 21.29, 28.26 and 37.23 g/100 g dry matter for the defatted flour, concentrate and isolate, respectively.

The compositions of essential amino acids of the flour, concentrate, and isolate of *C. marginella* were significantly different (*p* < 0.05). Methionine + cysteine was the abundant essential amino acid in the defatted flour while histidine and threonine were in the highest concentration in the concentrate. Valine, isoleucine, leucine, lysine, and phenylalanine + tyrosine were found in the highest concentration in the isolate. Threonine, methionine + cysteine, isoleucine, leucine and lysine contents of isolate fractions of *C. butyrospermi* were significantly higher than those in the protein concentrate and defatted flour while valine and phenylalanine + tyrosine were in the highest content in the protein concentrate and defatted flour, respectively.

**Fig. 3:**
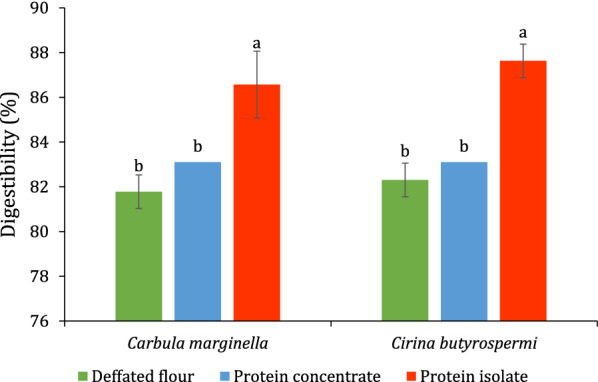
Digestibility (%) of defatted flour, protein concentrate and isolate of *Carbula marginella* and *Cirina butyrospermi*

The isolate fractions of both *C. marginella* (86.57 ± 0.75%) and *C. butyrospermi* (87.63 ± 0.75%) exhibited the highest protein digestibility (Fig. 3). The lowest digestibility was recorded for the defatted flour and protein concentrates for both *C. marginella* and *C. butyrospermi* which were not significantly different too.

**Fig. 4:**
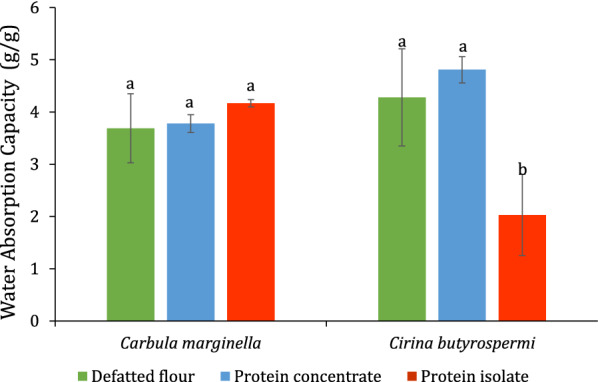
Water absorption capacity (g/g) of defatted flour, protein concentrate and isolate of *Carbula marginella *and *Cirina butyrospermi*

The water absorption capacity was comprised between 2.03 and 4.81 g/g for the protein isolates of *C. butyrospermi* and the protein concentrates of *C. butyrospermi*, respectively (Fig. 4). No significant difference was observed between the defatted flours and the protein concentrates of both *C. marginella* and *C. butyrospermi*, and the protein isolates of *C. marginella* while the protein isolates of *C. butyrospermi* was different from the others.

**Fig. 5:**
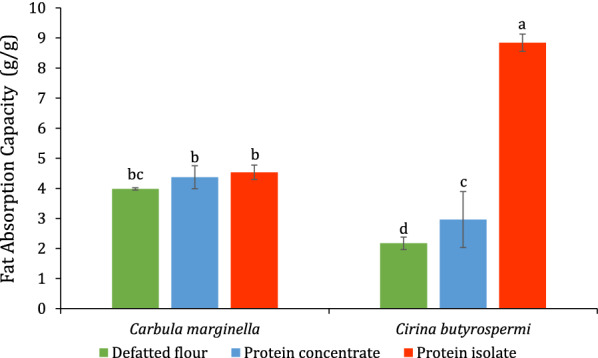
Fat absorption capacity (g/g) of defatted flour, protein concentrate and isolate of *Carbula marginella* and *Cirina butyrospermi*

The highest (8.84 ± 0.29 g/g) and the lowest (2.17 ± 0.21 g/g) fat absorption capacities were obtained with the protein isolates and the defatted flour of *C. butyrospermi*, respectively (Fig. 5). The fat absorption capacities of the defatted flour, the protein concentrates, and isolates of *C. marginella* were not significantly different (*p* < 0.05).

**Fig. 6:**
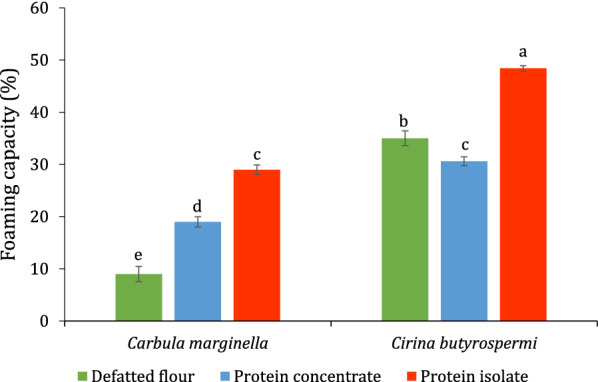
Foaming capacity (%) of defatted flour, protein concentrate and isolate *Carbula marginella *and* Cirina butyrospermi*

The protein isolate of *C. butyrospermi* had the highest foaming capacity (48.40 ± 0.56%) while the lowest was obtained for the defatted flour of *C. marginella* (9.00 ± 1.47%) (Fig. 6). Foaming capacities of the defatted flour, the protein concentrates, and isolates of both *C. marginella* and *C. butyrospermi* were significantly different (*p* < 0.05).

## Discussion

### Moisture contents

The moisture contents on the fresh basis of both *C. marginella* and *C. butyrospermi* (Table 1) were in the range of those reported for *Oecophylla smaragdina*, *Odontotermes *sp., *Hermetia illucens *L., *Apis mellifera ligustica*, and *Musca domestica L.* [31–33]. These high moisture contents make them sensitive to rapid degradation. This could explain why they are immediately processing by boiling and drying after collection.

### Ash and mineral contents

Minerals are essential for the metabolic processes of the human body. The ash contents of *C. marginella* and *C. butyrospermi* (Table 1) were lower and higher than the mean value of 5.03 and 4.51% reported for Hemiptera and Lepidoptera members, respectively [6]. The ash content of *C. marginella* was similar to that of *Neortholomus* sp [34]. *Cirina butyrospermi* had an ash content similar to that reported by Yapo et al. [17] in Côte d’Ivoire but lower than the 5.10% value already recorded in Burkina Faso [16] with the same species.

*Carbula marginella* had lower calcium, iron, magnesium, potassium, sodium, and zinc contents (Table 2) than that reported for *Agonoscelis pubescens*, *Aspongubus viduatus*, and *Euschistus *sp., members of *Hemiptera* orde*r* [34, 35]. Iron, potassium, and sodium contents of *C. butyrospermi* were higher than those previously reported in Burkina Faso and in Côte d’Ivoire while its calcium and magnesium contents were higher and lower than that found in Côte d’Ivoire and in Burkina Faso, respectively [16, 17]. The zinc content was lower and higher than the values previously reported by the same authors in Côte d’Ivoire and in Burkina Faso, respectively [16, 17]. Variations in these values could be due to analytical methods, insect feed, and geographical location. Fe and Zn contents obtained with *C. marginella* and *C. butyrospermi* covered the recommended dietary intake of Fe for adults [36]. Both *C. marginella* and *C. butyrospermi* can be used as an alternative source of Fe and Zn in the food supplementation program [37].

### Fat and fatty acid compositions

The fat contents of both *C. marginella* and *C. butyrospermi* (Table 1) were higher and lower than those reported for the members of Hemiptera and Lepidoptera orders, respectively [6]. The fat content of *C. butyrospermi* was lower and higher than that of 28.71 and 14.51% previously reported by Yapo et al. [17] and Anvo et al. [16], respectively.

The content of palmitic, oleic, and linoleic acids of *C. marginella* (Table 3) were, respectively, in the range of 2.47 to 31.80 ; 0.92 to 45.53 and 4.90 to 35.21 reported for *A. pubescens*, *A. viduatus, Lethocerus indicus*, and *Meimuna opalifera*, members of Hemiptera order [34, 35, 38, 39]. Compared to these members, *C. marginella* oil had a relatively balanced fatty acid composition which included 38.04% of saturated fatty acids, 30.79% of monounsaturated fatty acids, and 31.13% of polyunsaturated fatty acids.

The crude oil of *C. butyrospermi* was characterized by oleic, stearic, linolenic, and palmitic acids as abundant fatty acids (Table 3). This composition was different from those previously reported by Yapo et al. [17] and Anvo et al. [16] which described stearic (39.53–35.40%, respectively) and linolenic (23.89–35.82%, respectively) acids as the dominant ones. The crude oil of *C. butyrospermi* can be considered a good source of linolenic acid, which is an essential fatty acid.

### Protein contents and amino acid compositions

The crude protein content of the defatted flour of *C. marginella* was described in the range of 27–72% for the members of Hemiptera order [6]. The protein content of the flour of *C. butyrospermi* was lower than those of 55.41 and 62.74% previously reported by Anvo et al. [16] and Yapo et al. [17], respectively. However, this protein content remained in the range of other Lepidoptera larvae, including *Galleria mellonella* (41.25%), *Heliothis zea* (42.00%), and *Aegiale hesperiaris* (40.24%) [34, 40, 41].

The higher protein content of isolate for both *C. marginella* and *C. butyrospermi* compared to concentrate could be due to diverging extraction methods. The alkaline extraction-isoelectric precipitation method improves the protein content [42]. Thus, Mishyna et al. [10] also reported that alkaline and sonication-assisted extractions enhanced protein content in *Schistocerca gregaria* powder by 14.8 and 19.4% respectively compared to raw powder. A similar increase was recorded for *A. mellifera*, where protein contents reached 39.6 and 55.2% for alkaline and sonication-assisted extractions. Wu et al. [43] also observed an increase in the protein content of the peanut protein isolate from 55.88% for peanut powder to 96.65% for the isolate. As legume protein isolates and concentrates that of *C. marginella* and *C. butyrospermi* can find applications in pasta and desserts industries [44–46].

With the exception of phenylalanine + tyrosine, all the essential amino acids in the Hemiptera order were lower than those of the flour of *C. marginella* [6]. The total essential amino acid content of the flour of *C. marginella* was higher than those reported by Mariod et al. [35] for *A. pubescens* and *A. viduatus*.

Except for valine, all the essential amino acids in the Lepidoptera order were lower than those of the flour of *C. butyrospermi*. The total essential amino acid content of the defatted flour of *C. butyrospermi* (Table 5) was lower than the value of 29.88 g/100 g dry matter previously reported by Anvo et al. [16]. Histidine, threonine, leucine, lysine, and phenylalanine + tyrosine contents of *C. butyrospermi* were lower than those reported by Anvo et al. [16] and Yapo et al. [17] for the same species. Variations in these values could be due to insect feeding, geographical location, and the different treatments undergone by the insects after collection. The protein concentrates and isolates of *C. marginella* and *C. butyrospermi* had lysine contents higher than those recommended by the FAO for the daily requirement of adults [44]. Cereals and pulses are characterized by low contents of Lysine and Methionine + cysteine, respectively [45, 46]. The high values of lysine and methionine + cysteine in concentrates and isolates of both species suggest that they can be used as dietary supplements in cereal and legume-based foods.

### Carbohydrates and energy values

The carbohydrate content of the defatted flour of both *C. marginella* and *C. butyrospermi* was described in the range of 0.01–26% and 1–66.10% for the member of Hemiptera and Lepidoptera orders, respectively [6]. The carbohydrate content of the flour of *C. butyrospermi* was higher than that reported by Yapo et al. [17] in Côte d’Ivoire and Anvo et al. [16] in Burkina Faso.

The energy value of *C. marginella* (650.23 ± 2.11 Kcal/100 g) was higher than the range of 328.99–622 Kcal/100 g described for the members of Hemiptera order [6]. It was also higher than that found by Durst et al. [41] with *Neortholomus* sp. (542.08 Kcal/100 g) and *Edessa petersii* (530 Kcal/100 g). The energy value of *C. butyrospermi* (480.20 ± 0.49 Kcal/100 g) was in the range of 293–776.85 Kcal/100 g described for the members of Lepidoptera order [6]. It was higher than that reported by Anvo et al. (432 Kcal/100 g) [16] but lower than that obtained by Yapo et al. [17] (492.31 Kcal/100 g) with the same species.

### Protein digestibility

Protein quality in food is determined by amino acid profile and the ability of digestive enzymes to liberate the amino acids [47]. The protein digestibility of the defatted flour of *C. marginella* and *C. butyrospermi* (Fig. 3) was similar to that reported for *Ruspolia differens* (82.34%), *Cirina forda* (81.71%), *Gryllus assimilis* (80.82%) and lower than the values of 84.98%, 85.67%, 83.41–90.49%, 90.66% reported for *Macrotermes nigeriensis, Ruspolia differens, Macrotermes subhyalinus*, and *Melanoplus foedus, respectively* [7]. The insect exoskeleton contains chitin which could lower its digestibility [48]. The high digestibility of isolate and concentrate fractions could be explained by the elimination of the chitin.

### *Functional properties*

Water absorption capacity (WAC) is the amount of water that can be bound or retained by proteins [49]. The water absorption capacity of defatted flour and concentrate of both C*. marginella and C. butyrospermi* (Fig. 4) was higher than those of the defatted flour of *C. forda* [50], the defatted flour of *Imbrasia oyenmensis* [51], and the concentrate of *Gryllodes sigillatus* [9]. The water absorption capacity of *C. butyrospermi* isolate was lower than that of the soy isolate [52] but higher than that of the isolate of peanut [43]. The highest values of water absorption capacity of the defatted flour and the concentrate of both *C. marginella* and *C. butyrospermi* could be due to the high content of hydrophilic amino acids [9]. The fat absorption capacity (FAC) is defined as the amount of fat retained by the proteins. It is also the sum of lipids bound by hydrophobic interactions between the protein and the fat itself, and the physically trapped fat in the protein matrix [49]. The defatted flour and the concentrate of *C. butyrospermi* showed lower FAC values than the defatted flour of *Acheta domesticus* and *G. sigillatus* [9, 53]. The FAC values of the isolate of *C. butyrospermi* were twofold higher than the highest value of 3.58 g/g reported in the literature [54]. The FAC is related to protein content, types of proteins, and the amino acid composition of proteins, especially to hydrophobic residues that interact with hydrocarbon chains in fat molecules [55]. The high FAC value of the isolate of *C. butyrospermi* could be due to the high content of non-polar amino acids which play an important role in the mechanism of oil absorption [9]. This is important since oil acts as a flavor retainer and increases the palatability of foods [56]. Foams are gas dispersions in a continuous phase that is usually a liquid [57]. Their formation requires the solubilization of the proteins in the aqueous phase and their rapid unfolding to form a cohesive layer of proteins around the gas/air droplets [58]. The foaming capacity of the defatted flour and the concentrate *C. butyrospermi* (Fig. 6) were in the range of that reported for the flour and the protein concentrate of *Tenebrio molitor* [9] while that of its isolate was similar to that of the protein concentrate of *A. mellifera* [10]. The lower foaming capacity of the defatted flour, the protein concentrates, and isolates of *C. marginella* compared to that of *Ci. butyrospermi* may be due to the physicochemical properties of their proteins [9]. Functional properties of protein products are physicochemical indicators that determine the behavior of proteins in the production of food products. These properties are mainly related to the structure and amino acid composition of native proteins. [54]. Foods with high water absorption capacities are used in the preparation of viscous foods, such as soups, sauces, pasta, and baked foods [59]. The protein concentrates and isolates of *C. marginella* could be used in the preparation of these types of foods. Knowledge of fat absorption capacity is important in food technology as it imparts certain characteristics to the product, such as flavor retention, palatability enhancement and increase in shelf life by reducing humidity and fat loss [60]. This property is mainly used in many food applications such as the meat and bakery industries [9]. Therefore, Protein concentrates and isolates of *C. marginella* and *C. butyrospermi* could be used in these industries due to their high oil absorption capacity.

## Conclusion

The study showed that both *C. marginella* and *C. butyrosperm*i are potential sources of protein, fat, and ash. Both insects were also excellent sources of iron, zinc, calcium, potassium, and essential fatty acids. Moreover, the protein concentrates and isolates of both species showed good technological properties. Both *C. marginella* and *C. butyrospermi* can therefore be used in the strategies to fight against protein-energy malnutrition and micronutrient deficiencies.

## Data Availability

The datasets used and/or analyzed during the current study are available from the corresponding author on reasonable request.

## Abbreviations

AOAC: Association of Official Analytical Chemists
Ca: Calcium
FA: Fatty acid
FAC: Fat absorption capacity
FAME: Fatty acid methyl esters
FC: Foaming capacity
Fe: Iron
GC: Gas chromatography
HPLC: High-performance liquid chromatography
IUPAC: International Union of Pure and Applied Chemistry
K: Potassium
Mg: Magnesium
MUFA: Monounsaturated fatty acids
Na: Sodium
PTFE: PolyTetraFluoroEthylene
PUFA: Polyunsaturated fatty acids
SFA: Saturated fatty acids
WAC: Water absorption capacity
Zn: Zinc
